# HIV testing and antiretroviral therapy initiation at birth: Views from a primary care setting in Khayelitsha

**DOI:** 10.4102/sajhivmed.v16i1.376

**Published:** 2015-04-28

**Authors:** Aurélie Nelson, Jean Maritz, Janet Giddy, Lisa Frigati, Helena Rabie, Gilles van Cutsem, Tabitha Mutseyekwa, Nomfusi Jange, Jonathan Bernheimer, Mark Cotton, Vivian Cox

**Affiliations:** 1 Médecins Sans Frontières, Khayelitsha, Cape Town, South Africa; 2 Department of Medical Virology, National Health Laboratory Service, University of Stellenbosch, Tygerberg Campus, South Africa; 3 Western Cape Department of Health, Khayelitsha and Eastern Substructure, Cape Town, South Africa; 4 Department of Paediatrics and Child Health, University of Stellenbosch, Tygerberg Campus, South Africa; 5 Médecins Sans Frontières, Cape Town, South Africa; 6 Centre for Infectious Disease Epidemiology and Research, University of Cape Town, South Africa

## Background

Despite 95% coverage via the prevention-of-mother-to-child-transmission (PMTCT) programme, 14 000 children in South Africa became HIV-infected in 2012.^1^ There are many reasons for this gap in PMTCT efforts. Late maternal diagnosis of HIV, late antenatal antiretroviral therapy (ART) initiation, seroconversion in pregnancy, and inadequate adherence to ART during gestation singly and collectively increase the risk of transmission. Early initiation of ART in the first few weeks of life significantly reduces the HIV-associated morbidity and mortality in HIV-infected infants, compared with deferred initiation^2,3^ and may reduce the latent HIV-1 reservoir in children.^4^ Furthermore, a recent South African study documents that 76% of babies who would have tested HIV positive via polymerase chain reaction (PCR) by 6 weeks could have been diagnosed at birth.^5^ Therefore, the Western Cape Province (WCP) Department of Health adopted new guidelines^6^ in June 2014 (and the South African Department of Health in December 2014^7^) to test ‘high-risk’ infants at birth. Infants testing PCR-positive are started on ART as soon as possible.

In March 2014, Médecins Sans Frontières, in collaboration with the University of Stellenbosch, started a pilot project in a community healthcare centre in Khayelitsha to establish the impact of very early infant diagnosis on ART initiation and to establish the feasibility of implementation in a primary care setting. To establish routine testing of high-risk HIV-exposed infants at the primary care level, the following steps were taken:

- training and mentorship of healthcare staff at the healthcare centre (midwives, nurses, doctors, counsellors) on the new guideline recommendations; the identification and management of high-risk infants; and the clinical management of ART initiation in a newborn
- routine counselling and consent from mothers for birth PCR testing
- initiation of nevirapine (NVP) and zidovudine (AZT) post-exposure prophylaxis on day one in high-risk infants
- PCR and confirmatory viral load testing via the routine courier system to the referring National Health Laboratory Service (NHLS) laboratory, with results available within 48 hours
- counselling all mothers regarding the PCR results at 48 hours of life, when the mother and infant return to the health centre for routine post natal care:
  ■ if the PCR result is negative, the mother is advised of the necessity to keep the infant in care for repeat HIV testing as per WCP guidelines as well as to continue post-exposure prophylaxis
  ■ if the infant is HIV positive, the nurse caring for the mother/infant pair provides standardised ART initiation counselling and ensures the infant is seen by a medical officer the same day
- initiation of ART on the same day as diagnosis for clinically well infants, according to interim infant ART treatment guidelines developed in consultation with infectious disease paediatricians, and referral to the district hospital for clinically unwell infants
- close follow-up of the infants on ART: weekly until 6 weeks of age and monthly until 2 years of age, including routine monitoring bloods and a repeat viral load at 4 months of age
- monitoring of further HIV test results in HIV-exposed infants testing negative at birth, to identify further HIV-positive infants and to assess the coverage of HIV testing per guidelines to 18 months of age.

By the end of December 2014, we had identified 138 high-risk infants delivered at the primary healthcare centre and we tested 132 high-risk infants (95% of high-risk infants delivered at the centre had a birth PCR) of whom 3 had a positive HIV PCR. The 3 cases are summarised in Table 1 and the lessons learnt from them are described below.

**TABLE 1:** Demographics and early outcomes of three HIV-positive infants diagnosed at birth in a primary care setting.

Demographics and outcomes	Case 1	Case 2	Case 3
Antenatal risk factors for transmission	Mother unbooked in labour (mother known ART treatment interrupter)	ART < 12 weeks in pregnancy (late booking at 25 weeks [HIV negative] and seroconverted at 32 weeks)	ART < 12 weeks in pregnancy (late booking at 31 weeks)
	–	–	Mother is a treatment interrupter
	–	–	Infant born prematurely at 36 weeks’ gestation
Mother's results	Viral load (2 weeks after delivery): 484	Viral load (2 weeks after delivery): 302	Viral load not available
	CD4 162 on day of delivery	CD4 281 at 32 weeks	CD4 293 at booking at 31 weeks
Time from birth to ART initiation	16 days	2.5 days	2 days
WHO stage at initiation	Stage 2	Stage 1	Stage 1
Challenges at initiation	Fear of disclosure and stigma	Low socio-economic status	Low socio-economic status
	Distrust of healthcare system	Financially dependent on partner	No stable relationship
	Poor social support	–	–
	Low socio-economic status	–	–
	Financially dependent on partner	–	–
**At ART initiation**			
Viral load (copies/mL)	871 740	1015	Lower than detectable (LDL) (repeat at 10 days of life-on ART: 265)
CD4 cell count (percentage of total lymphocytes)	1731 (34%)	2082 (27%)	1364 (66%)
HIV drug resistance testing	No resistance	Amplification not possible owing to low viral load	Amplification not possible owing to low viral load
Feeding method	Formula feeding	Reverted to breast feeding when infant initiated ART	Reverted to breast feeding when infant initiated ART
First ART regimen	AZT/3TC/NVP	AZT/3TC/NVP	AZT/3TC/NVP
Second ART regimen: NVP replaced with LPV/r	3 weeks of age (gestational age unknown)	2 weeks of age (gestational age unknown)	6 weeks of age (42 weeks’ gestational age)
Prophylactic medications	Co-trimoxazole (started at 4 weeks of age)	Co-trimoxazole (started at 4 weeks of age)	Co-trimoxazole (started at 4 weeks of age)
	Isoniazid (INH) for 6 months	–	–
Viral load (copies/mL) at 4 months†	1823	< 40 (month 3)	< 40
	-	AZT replaced by ABC	AZT replaced by ABC
Viral load (copies/mL) at 8 months	< 40	Not available yet	Not available yet
	AZT replaced by ABC	–	–
Mother VL 3–4 months after delivery	< 40	< 40	< 40
Adverse events	Hospitalised for bronchiolitis and to exclude TB (owing to presumed TB contact). Completed 6 months of INH.	Mild anaemia (Hb 10.4)	Hospitalised for initial high ALT at ART initiation (probably lab error). Repeat was normal.
Other challenges encountered	Transient migration to Eastern Cape over holiday period	Initial mix-up with ARV dosage despite extensive counselling	Mother claimed to be ART naïve at booking
	–	Transient migration to Eastern Cape over holiday period	–

†, Viral load recommended at 4 months after ART initiation as per Western Cape Government Department of Health prevention-of-mother-to-child-transmission clinical guidelines update.^6^

ART, antiretroviral therapy; NVP, nevirapine; AZT, zidovudine; INH, Isoniazid.

## Cases of positive HIV polymerase chain reaction: Lessons learnt

### Case 1

Case 1 was complicated by a mother struggling with numerous psychosocial difficulties: low socioeconomic status, fear of stigmatisation and of disclosure to her partner/family, and deep mistrust of the healthcare system. Despite repeated attempts to contact her, she only returned to care when the infant was 9 days old after her perception of her baby being healthy changed after the infant developed conjunctivitis. This case was a good illustration of how barriers at the individual level (low socioeconomic background and psychological factors such as fear of disclosure and stigma) can prevent access to ART for the infant.^8^ By addressing these barriers through individual counselling by healthcare providers and peer support from an organisation experienced in supporting HIV-positive mothers, the mother and infant returned to care and have remained in care to date (10 months later). They are both showing good adherence and viral suppression.

### Case 2

Case 2 confirmed the higher risk of transmission with a presumed HIV incident infection in the mother, as reported in other studies such as that by Drake et al.^9^ Good support and counselling allowed the mother to overcome her initial shock and to accept the need to initiate her infant on ART. She and her baby are suppressed and doing very well.

### Case 3

Case 3 illustrates the importance of early booking as intrauterine HIV transmission to the infant could have been prevented if the mother had been diagnosed and started on ART earlier in the pregnancy. In this instance, too, extensive support and counselling assisted the mother in accepting the diagnosis and starting treatment for the infant. Both are now virologically suppressed.

So far, we have demonstrated that testing high-risk infants at birth is possible in a primary care setting, with 129 out of 130 mothers eager for their infants to be tested at birth (other high-risk infants were not tested for other reasons such as already being enrolled in another study, mother missed, etc.). Although concerns were expressed by healthcare providers and counsellors on the psychosocial readiness of the mothers to initiate ART, we found that it was possible and sustainable, with structured ART initiation counselling and adherence support. ART programmes aimed at the implementation of early infant diagnosis strategies should consider modification of the existing three pre-ART routine counselling sessions as essential, given the timing of ART initiation in newborns and the early challenges that both mother and infant are likely to experience in the first months on ART.

Prior to starting the pilot study, we had concerns about the timing of sample transport from the primary care setting to the central laboratory. In fact, the average turnaround time for PCR results has been 48 hours, allowing prompt initiation of babies on ART after birth. Programmatically, it may be more difficult in rural areas, where there may be a longer turnaround time to obtain results. Furthermore, in our pilot project, about 22% of women did not come back for the results of the baby's birth PCR. Using a point of care (POC) PCR machine at birth, with good sensitivity and specificity, could reduce the attrition of patients not returning for results and improve early management of HIV-exposed infants. More research is needed to look into which POC platform would be optimal and the feasibility of its use at a primary care level.

ART initiation in this pilot project was undertaken by a general medical officer in a primary care setting, with the telephonic support of infectious disease paediatricians. As described in an article by Nuttall^10^ in the present issue of the *Southern African Journal of HIV Medicine*, initiating ART in a neonate is more intricate than for a 6-week-old infant in terms of available drugs, dosage and monitoring. An initial lack of confidence to initiate neonates on ART was observed during the mentorship of primary care medical officers, mostly related to lack of experience in calculating paediatric dosages and in blood draws in neonates. With the guidance of infectious disease paediatricians at our tertiary referral centre, we designed an interim guide on ART initiation at birth where we attempted to simplify blood tests for monitoring and the calculation of dosages. Modelled on existing ART drug dosing charts, the guide reduces the complexity of neonatal ART initiation and thus increases the confidence of clinicians managing well infants on ART at primary care level.

In addition to being well received by primary care doctors, mentorship on primary care ART initiation for stable infants was well received by the local district hospital, as it reduces the burden on their overstretched services. Furthermore, from the patient's point of view, previous studies show that accessing local clinics rather than distant referral hospitals leads to better retention in care,^8,11^ which is what we experienced on the ground, with the above three patients’ mothers being compliant with their appointments. The mother builds a trusting relationship with one or two health workers, which probably additionally promotes her retention in care. As we observed, assisting the very vulnerable mother-infant pair through the initial phase of ART may greatly assist long-term compliance with clinic visits.

The major difficulty encountered with ART initiation was for the mother to understand the dosage of syrups appropriately, particularly with the high number of changes in the first few months of treatment. This finding is corroborated by other MSF projects in which paediatric treatment failure has been linked directly to under-dosage by the caregiver (internal source). To overcome this difficulty, sufficient time must be spent counselling the mother on how to administer syrups; visual methods on how much of each syrup to give (using colour stickers and marked syringes) can be very helpful for mothers to understand the measurements. Assigning specific responsibility to the pharmacist, counsellor, nurse or doctor for this education on medication dosing is crucial for ART initiation in neonates to be rolled out effectively in a primary care setting.

Finally, whilst following up the birth PCR-negative babies, we observed that most women take their infants for a ‘6-weeks PCR’ but very few of them actually come to clinics in the right time frame (6–8 weeks). Most of the ‘6-weeks PCR’ tests are done at random times, showing that mothers return to clinics when it suits them, not when they are given a date. Further studies are needed to evaluate the impact of this observation as well as the reasons behind it, and how to adapt the existing system to the needs and demands of new mothers.

## Conclusion

We started a pilot project in a primary care centre in Khayelitsha in March 2014 to establish the impact of very early infant diagnosis in reducing the age at ART initiation, and to investigate the feasibility of implementing both early diagnosis and early ART initiation in a primary care setting. So far, we have diagnosed 3 HIV-positive infants out of 129 high-risk HIV-exposed babies tested, and all 3 babies were initiated on ART from 2 days to 2.5 weeks after birth. They are currently doing well: all 3 infants are adherent to their medication and are virologically suppressed on ART. Overall, we have established the feasibility of testing newborns for HIV at birth and initiating ART in the first days of life in a primary care setting with appropriate mentorship of healthcare providers and consistent adherence support for mothers. By promoting a decentralised model of early infant diagnosis at birth, programmes will ensure access to care for some of the most vulnerable patients with HIV infection.
